# A Case of Cervical Intraneural Lipoma That Was Removed by Intercapsular Resection with No Resultant Postoperative Neurological Deficit

**DOI:** 10.1155/2022/4618731

**Published:** 2022-06-20

**Authors:** Hitoshi Sato, Yoshiro Saito, Tatsuya Kitajima, Shunya Egawa, Toshikazu Shimane

**Affiliations:** ^1^Department of Oral and Maxillofacial Surgery, Division of Oral Oncology, Showa University, School of Dentistry, 2-1-1 Kitasenzoku, Ohta-Ku, Tokyo 145-8515, Japan; ^2^Showa University Head and Neck Oncology Center, 1-5-8 Hatanodai, Shinagawa-Ku, Tokyo 142-8555, Japan

## Abstract

Intraneural lipomas in peripheral nerves of cervical lesions are extremely rare and have not been previously reported. We present a 48-year-old male with a gradually increasing right cervical mass since 5 years. He visited our department because of pain and difficulty in raising the right upper limb. A tumor about 80 mm in size was palpable in the right neck along the cervical nerve. The tumor was suspected to involve fatty degeneration in schwannoma of cervical nerve origin, for which intercapsular resection was performed under general anesthesia. Histopathologically, bifurcated growth of mature adipocytes with sparse fibrous septa and lack of tumor proliferation of Schwann cells was observed on H&E staining, suggesting a diagnosis of intraneural lipoma. The patient had no new motor or sensory deficits postoperatively and with improvement in his preoperative symptoms.

## 1. Introduction

While lipomas in the cervical region are common, intraneural lipomas in peripheral nerves in this region are extremely rare. Although few cases of intraneural lipomas in the upper limbs have been reported by orthopedists [1, 2], to the best of our knowledge, there are no reports of such tumors in the cervical region. We describe here a case that was initially suspected as schwannoma of cervical nerve origin that was finally diagnosed as a cervical intraneural lipoma based on lack of histopathological evidence of schwannoma.

### 1.1. Case Presentation

**Figure 1:**
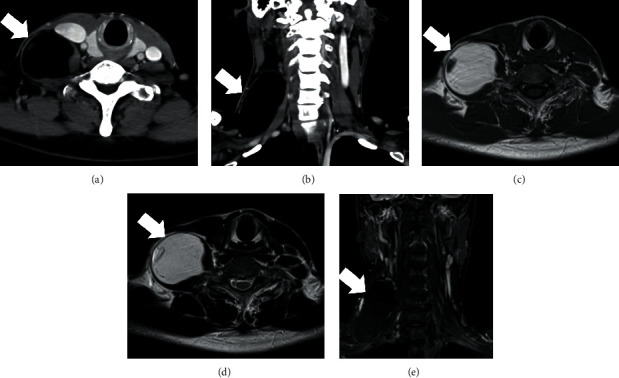
Imaging findings: contrast-enhanced computed tomography ((a) horizontal section and (b) coronal section) showing a well-defined, 85 × 50 mm tumor (arrow) in the right neck. There was no contrast effect inside the tumor. Contrast-enhanced MRI ((c) T1-weighted images, (d) T2-weighted images, and (e) STIR) shows the tumor had low signal intensity on T1-weighted images, high signal intensity on T2-weighted images, and low signal intensity on STIR. Variable signal intensity areas were seen outside the tumor.

**Figure 2:**
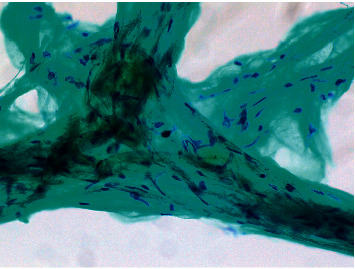
Fine needle aspiration cytology findings (Papanicolaou stain). There were a small number of mesenchymal cells with spindle-shaped nuclei.

A 48-year-old Japanese man with unremarkable medical and family histories presented with a gradually increasing mass on the right side of the neck since 5 years, causing pain and difficulty in raising the affected upper limb. Examination showed an elastic soft tumor on the right side of the neck, measuring about 80 mm along the cervical nerve from the hyoid bone to the level of the clavicle. Although the patient experienced pain and difficulty in raising the right arm, there was no clear evidence of neurological deficits due to tumor compression. Contrast-enhanced computed tomography (CT) showed a well-defined 85 × 50 mm tumor on the right neck (Figures 1(a) and 1(b)), with low radiodensity, equivalent to that of adipose tissue. There was no contrast effect inside the tumor, although it showed partial enrichment that was different from the surrounding areas. Contrast-enhanced MRI showed a well-defined right neck tumor with high signal intensity on T1-weighted images, high signal intensity on T2-weighted images, and low signals on STIR (Figures 1(c)–1(e)). Some flat areas of variable signal intensity were seen outside the tumor. Fine needle aspiration cytology (FNAC) showed a small number of mesenchymal cells with spindle-shaped nuclei (Figure 2), consistent with a schwannoma.

**Figure 3:**
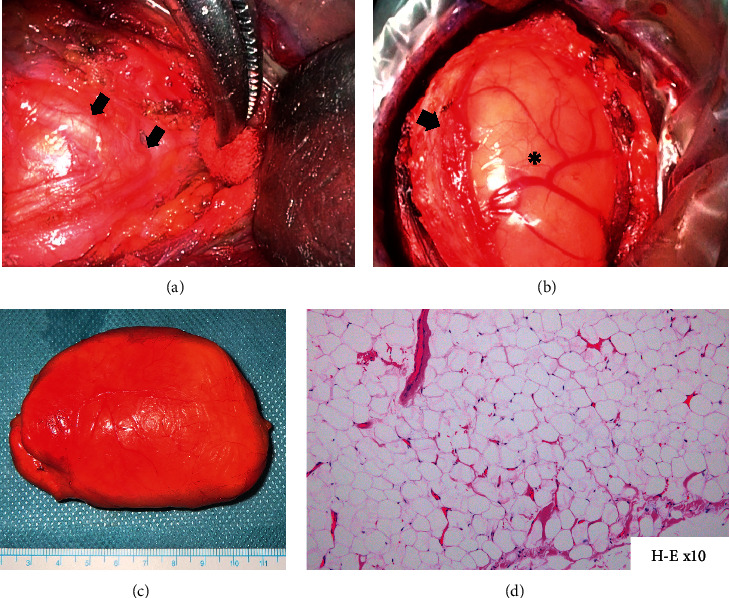
Intraoperative findings: the nerve of origin at the lower edge of the tumor ((a) arrow) and tumor in the neuroepithelium (arrow, epineurium; (b)^*∗*^, tumor) were visible. The excised yellowish specimen was 85 × 50 × 35 mm (c). Histopathologically, bifurcated growth of mature adipocytes with sparse fibrous septa was observed (d) (H&E staining).

Although lipoma was suspected by imaging findings, the variable signal areas within the tumor on MRI, FNAC results, and location and course of the tumor were consistent with schwannoma of cervical nerve origin. Thus, we clinically diagnosed the tumor as fatty degeneration of cervical nerve schwannoma and performed surgical excision under full informed consent, including explanation of the possibility of postoperative neurological deficits. Under general anesthesia, a transverse incision was made just above the tumor and subcutaneous dissection was performed confirming inclusion of the entire tumor. The peritumor area was dissected from the anterior border of the sternocleidomastoid muscle, and the nerve of origin and its course were identified at the superior and inferior edges of the tumor (Figure 3(a)). The nerve was dorsal to the tumor, and since no response to stimulation of the anterior surface of the tumor using a nerve stimulator was observed, the epineural incision was made anteriorly. The fibrous connective tissue was dissected to reach the true fatty and yellowish tumor capsule (Figure 3(b)). The tumor was removed by intercapsular dissection in a cephalocaudal direction, preserving the originating nerve. The tumor was easy to remove as it was softer than most schwannomas and there was less blood loss. The epineurium was then brought together to ensure hemostasis, and a continuous suction drain was placed to complete the procedure. The operation lasted 3 hours and 33 minutes, with minimal blood loss. There were no new motor or sensory deficits postoperatively. The preoperative difficulty in raising the affected upper limb and pain disappeared by the time of the patient's discharge. Histopathological examination of the excised yellowish specimen (85 × 50 × 35 mm; Figure 3(c)) revealed bifurcated growth of mature adipocytes with sparse fibrous septa on H&E staining (Figure 3(d)). Similar findings were also observed in what was thought to be the enriched component on preoperative imaging (Figure 3(d)).

## 2. Discussion

Schwann cells, from which schwannomas develop, are glial cells that surround the axons of peripheral nerve cells, bundling some nerve cell axons and forming a myelin sheath around them. Myelinated fibers are encapsulated by the endoneurium. Nerve bundles are composed of many similar fibers and are encapsulated by the perineurium. Other nerve bundles, blood vessels, and fat, wrapped in the epineurium, form a nerve structure. Both schwannomas and intraneural lipomas arise within the epineurium, making it difficult to differentiate between them by preoperative imaging. Fatty degeneration of schwannomas is rare and has not been reported in the head and neck region [3]. In our experience of 100 cases of cervical schwannomas from 2005 to 2019, none of them developed fatty degeneration [4] nor have we experienced a case that was diagnosed as intraneural lipoma after surgery for suspected schwannoma, as in this case [3]. In the present case, the presence of spindle-shaped cells on FNAC increased the suspicion of schwannoma, although final histopathology showed only mature fat cells. This histopathological finding was seen in what was thought to be the area of enrichment on preoperative imaging. The reason for the presence of spindle-shaped cells on FNAC could be that cells from the epineurium and its surroundings were aspirated during FNAC of the intraneural tumor. As a peripheral nerve structure, although the neuroepithelium contains blood vessels and fatty tissue, lipomas originating from this fatty tissue are extremely rare. Although there are few reports of this tumor in the upper limbs, we did not find any previous reports of such tumors in the head and neck region, suggesting the uniqueness of this case. In 1981, Rusko et al. [1] described intraneural lipoma as a fatty tumor arising in or around a peripheral nerve and consisting of mature fat cells with a coating that was detachable from the nerve tissue and histopathologically free of nerve cells. Later, Fletcher et al. [5] described lipomatosis of the nerve as a group of various histological types, with abnormal proliferation of adipose tissue occurring inside or outside the neuroepithelium as the true definition of intraneural lipoma, as proposed by Rusko [1]. Recently, some reports have confused intraneural lipoma and lipomatosis of the nerve, and there is also a hybrid type of lipomatosis, in which fatty tissue envelops the nerve from the outside, and intraneural lipoma, in which fatty tissue proliferates within the neuroepithelium [6]. In the present case, intraoperative evaluation showed lipoma located below the epineurium, and the histopathological findings also revealed a true intraneural lipoma. Lipomatosis of the nerve, other than intraneural lipoma, is also a rare disease. Lipomatosis of the nerve is a soft, slowly growing tumor that presents with neurological symptoms as it enlarges. It occurs at a relatively young age and has been reported to be less likely to enlarge after the age of 30–40 years. It affects both sexes equally and most frequently involves the median nerve, followed by the ulnar and plantar nerves, among other sites [7]. Its incidence is 78–96% in the upper extremities and 4–22% in the lower extremities [8]. Recently, it has been reported that activation of PIK3CA mutations is a frequent event in lipomatosis of the nerve, irrespective of the affected peripheral nerve or the presence or absence of overgrowth of nerves in unaffected areas [9]. Due to the phenotypic diversity of PIK3CA-related overgrowth spectrum, Blackburn et al. [10] proposed the term “PIK3CA-related lipomatosis of the nerve” for this entity. Although the frequency of PIK3CA mutations raises the possibility of efficacy of PI3K/AKT/mTOR-targeted therapies for lipomatosis of nerves, the frequency of these mutations in intraneural lipomas is unclear because of the rarity of the tumor. In future, accumulation of more cases of intraneural lipomas and their genetic analysis are needed for their further evaluation.

True intraneural lipomas are typically treated with total resection of the tumor within the epineurium, while combined resection of the nerve and tumor is performed for lipomatosis of the nerve, with subsequent nerve reconstruction [6]. Decompression alone is sometimes performed because of the slow growth of lipomatosis of the nerve [6]. In the present case, intercapsular resection was performed due to the clinical suspicion of schwannoma. Fortunately, the tumor was removed within the tumor capsule. The tumor capsule is what is the epineurium which is including nerve fibers covered with perineurium. Theoretically, because intraneural lipoma which originated from the adipose within the epineurium never adheres directly nerve fibers, intercapsular resection of intraneural lipoma such as the present case would not occur postoperative neurological deficit. Our experience suggests that careful surgical procedures through the epineurium are necessary to reduce the risk of transient or permanent motor and sensory disturbances due to nerve damage.

## 3. Conclusion

We performed intercapsular resection of an intraneural lipoma, based on a preoperative diagnosis of fatty schwannoma. Postoperative histopathological evaluation revealed intraneural lipoma based on lack of tumor proliferation of Schwann cells. Intraneural lipomas in the neck are extremely rare and have not been previously reported. Our patient's preoperative symptoms improved without any postoperative neurological deficits.

## Data Availability

The data used to support the findings of this study are included within the article.
